# Interpretation and application of carbon isotope ratios in freshwater diatom silica

**DOI:** 10.1002/jqs.2837

**Published:** 2016-06-17

**Authors:** Megan Webb, Philip A. Barker, Peter M. Wynn, Oliver Heiri, Maarten van Hardenbroek, Frances Pick, James M. Russell, Andy W. Stott, Melanie J. Leng

**Affiliations:** ^1^ Lancaster Environment CentreUniversity of LancasterLancasterUK; ^2^ Institute of Plant Sciences and Oeschger Centre for Climate Change ResearchUniversity of BernBernSwitzerland; ^3^ Geography and EnvironmentUniversity of SouthamptonSouthamptonUK; ^4^ STREAM Industrial Doctorate CentreUniversity of SheffieldSheffieldUK; ^5^ Department of Earth, Environmental and Planetary SciencesBrown UniversityProvidenceRIUSA; ^6^ Stable Isotope FacilityNERC Centre for Ecology & Hydrology, Lancaster Environment CentreLancasterUK; ^7^ NERC Isotope Geosciences FacilitiesBritish Geological SurveyNottinghamUK; ^8^ Centre for Environmental GeochemistrySchool of GeographyUniversity of NottinghamNottinghamUK

**Keywords:** carbon cycling, diatom frustule carbon, Lake Tanganyika, palaeoclimate, stable carbon isotopes

## Abstract

Carbon incorporated into diatom frustule walls is protected from degradation enabling analysis for carbon isotope composition (δ^13^C_diatom_). This presents potential for tracing carbon cycles via a single photosynthetic host with well‐constrained ecophysiology. Improved understanding of environmental processes controlling carbon delivery and assimilation is essential to interpret changes in freshwater δ^13^C_diatom_. Here relationships between water chemistry and δ^13^C_diatom_ from contemporary regional data sets are investigated. Modern diatom and water samples were collected from river catchments within England and lake sediments from across Europe. The data suggest dissolved, biogenically produced carbon supplied proportionately to catchment productivity was critical in the rivers and soft water lakes. However, dissolved carbon from calcareous geology overwhelmed the carbon signature in hard water catchments. Both results demonstrate carbon source characteristics were the most important control on δ^13^C_diatom_, with a greater impact than productivity. Application of these principles was made to a sediment record from Lake Tanganyika. δ^13^C_diatom_ co‐varied with δ^13^C_bulk_ through the last glacial and Holocene. This suggests carbon supply was again dominant and exceeded authigenic demand. This first systematic evaluation of contemporary δ^13^C_diatom_ controls demonstrates that diatoms have the potential to supply a record of carbon cycling through lake catchments from sediment records over millennial timescales.

**Abbreviations**
CCM: carbon concentrating mechanism
DIC: dissolved inorganic carbon
EC: electrical conductivity
TDN: total dissolved nitrogen
TN: total nitrogen
TP: total phosphorus

## Introduction

Stable isotope analyses of the siliceous cell walls (frustules) of diatoms provide insights into a broad range of environmental processes tracked from the perspective of a single, ecologically well‐constrained organism. To date, most diatom‐based stable isotope studies have focused on the stable oxygen and silicon isotope composition (δ^18^O_diatom_ and δ^30^Si_diatom_) of diatoms from lacustrine and marine sediments (Leng and Barker, 2006; Swann and Leng, 2009; Leng and Henderson, 2013). Changes in δ^18^O_diatom_ are used as a proxy record of water source and hydrological balance in palaeolimnology (Barker *et al*., 2001; Rioual *et al*., 2001; Shemesh *et al*., 2001) and global ice volume, temperature and local effects in palaeoceanography (Shemesh *et al*., 1995, 2002; Hodell *et al*., 2001). δ^30^Si_diatom_ in freshwater is used to understand changes in climate, weathering and soil processes through the balance of silicon supply and demand (De La Rocha *et al*., 2000; Ding *et al*., 2004; Street‐Perrott *et al*., 2008). Within marine environments, utilization of dissolved silica can be reconstructed through the ratio of silicic acid uptake by diatoms to initial dissolved concentrations (De La Rocha *et al*., 1998; Varela *et al*., 2004; Cardinal *et al*., 2005).

Diatom frustules are also a host for carbon isotopes measured on organic molecules occluded within diatom frustule walls (δ^13^C_diatom_). This occluded organic matter comprises proteins and long‐chain polyamines (Kröger and Poulsen, 2008) and represents a source of carbon potentially protected from degradation over geological timescales (Singer and Shemesh, 1995; Crosta and Shemesh, 2002). During cell formation diatoms source this carbon via photosynthetic uptake from the surrounding water body. The fraction available for photosynthesis is dissolved inorganic carbon (DIC), which diatoms take up preferentially as dissolved CO_2_ or as bicarbonate under conditions of high carbon demand (Giordano *et al*., 2005). δ^13^C_diatom_ provides a record of changes in this carbon pool, overcoming issues of sample heterogeneity and potential diagenesis associated with investigations of stable carbon isotopes of bulk organic material (δ^13^C_bulk_) from sediments.

Use of δ^13^C_diatom_ as a palaeoenvironmental proxy is already well established within palaeoceanography where δ^13^C_diatom_ is usually interpreted as a record of pelagic primary productivity as discrimination against ^13^C by diatoms is reduced during periods of high carbon demand (Singer and Shemesh, 1995; Crosta and Shemesh, 2002; Schneider‐Mor *et al*., 2005). However, care is required in interpretation of δ^13^C_diatom_ records, as further biological variables with potential to impact carbon fractionation and isotope composition are yet to be fully constrained (Jacot Des Combes *et al*., 2008). These variables include diatom species assemblage, carbon availability and carbon source.

Two key factors that determine the degree of fractionation during photosynthetic carbon uptake are the balance between internal and external CO_2_ concentrations and discrimination by the enzyme RuBisCO (Jacot Des Combes *et al*., 2008). The impact of these factors is compounded by species‐specific ‘vital effects’, including cell growth rate, geometry and growth environment. For example, high cell growth rates reduce the internal to external CO_2_ ratio (Hill *et al*., 2008). Discrimination by RuBisCO to ^13^C is theoretically proportional to this ratio (Korb *et al*., 1996; Hill *et al*., 2008), and fractionation of carbon will subsequently be less in faster growing cells. Conversely, where cell geometry maximizes the surface area to volume ratio, CO_2_ is more efficiently absorbed leading to a relative increase in carbon fractionation (Popp *et al*., 1998). Planktonic species have also been associated with lower δ^13^C values compared with benthic varieties, attributed to the more turbulent growth environment of the former, which reduces the impact of boundary layer thickness on carbon uptake (France, 1995; France and Cattaneo, 1998; Wang *et al*., 2013).

Confinement of carbon isotopic analysis to the initial protein matrix established during cell formation represents a carbon source less likely to be affected by such species‐specific effects. In fact, tests of the impact of different diatom species composition on δ^13^C_diatom_ from freshwater Lake Challa, Mount Kilimanjaro, were within analytical error (Hurrell *et al*., 2011). To reduce the risk of any vital or species‐specific effects, it is recommended within the more established field of palaeoceanography to sieve to the <20‐μm fraction as it is here that most diatom material is found and assemblages tend to be dominated by fewer species (Crosta and Shemesh, 2002).

Availability of DIC is also a key determinant of carbon fractionation, as slow diffusion of dissolved CO_2_ through water risks transport limitation. To prevent this, carbon concentrating mechanisms (CCMs), which manifest as active uptake of dissolved CO_2_ and/or bicarbonate, are believed to take place in almost all diatoms (Giordano *et al*., 2005). Utilization of bicarbonate by diatoms can result in a further increase in δ^13^C of the photosynthate by approximately 9‰ (Finlay, 2004). Whilst interpreting δ^13^C_diatom_ records it is therefore important to consider whether an increase in value has been enhanced by carbon transport limitation.

Of particular relevance to interpretation of freshwater δ^13^C_diatom_ is the carbon isotope composition of DIC sources (δ^13^C_DIC_). A far greater variety of carbon sources is found within terrestrial environments compared with the open ocean. The associated δ^13^C_DIC_ is in turn diverse, ranging from 0 to +1‰ for carbonate bedrock and from −26 to −20‰ for soil carbon in C_3_ landscapes, for example (Clark and Fritz, 1997). The expression of these different origins is then modified by mixing and fractionation during carbon phase transformation and species changes in transfer from the catchment to the water body, or because of internal aquatic processing (Finlay, 2003).

As a possible consequence of greater complexity introduced by more diverse carbon sourcing, there have been far fewer studies of δ^13^C_diatom_ in palaeolimnology compared with palaeoceanography. Interpretations reached have also been inferential rather than reconstructions grounded in, and constrained by, modern environmental data (Hernández *et al*., 2011, 2013; Barker *et al*., 2013). For example, in Lago Chungará, Chile, diatoms deposited over the late glacial to early Holocene period have high δ^13^C_diatom_ values (−27.5 to −22.6‰) during arid stages compared with those of wetter, humid periods (−30.3 to −25.4‰) when greater input of ^13^C depleted dissolved biogenic carbon to the lake from the catchment was likely (Hernández *et al*., 2011, 2013). Similarly, a sediment core from Lake Challa, Mount Kilimanjaro, displayed positive correlation between δ^13^C_diatom_ and δ^13^C_bulk_ during dry intervals as high diatom productivity depleted the lake DIC pool. This correlation largely broke down during wetter periods and it was hypothesized that increased catchment carbon loading satisfied demand from primary productivity (Barker *et al*., 2013). These lake sediment records demonstrate an application for δ^13^C_diatom_ in tracing catchment carbon cycling, and the importance of testing the down‐core changes against contemporary environmental relationships.

Here we (i) explore relationships between water chemistry and δ^13^C_diatom_ from contemporary data sets to more precisely determine the environmental controls of δ^13^C_diatom_, and (2) apply the understanding gained to assess the utility of δ^13^C_diatom_ in unravelling temporal carbon dynamics. Firstly, diatom epilithon (diatoms extracted from submerged biofilm comprising other algae, bacteria, fungi and the products they secrete) from river reaches in north‐west England were sampled to provide an understanding of detailed carbon dynamics at the catchment scale. Rivers were investigated as they represent active hydrological pathways, connecting a lake with its catchment. Secondly, δ^13^C_diatom_ from sediment samples of lakes situated across central, north‐west and northern Europe were analysed. These sediments were collected as part of a broader sampling campaign designed to study the relationship between methane concentrations, δ^13^C of DIC and methane, and the carbon isotopic composition of aquatic invertebrate fossils and other sediment components (e.g. Schilder *et al*., 2015; Stötter *et al*., *unpublished data*). The lakes are geographically dispersed, spanning different climate zones ranging from temperate to boreal and incorporating diverse catchment geology with examples of calcareous and non‐calcareous lithology (Rinta *et al*., 2015). Thirdly, we applied the method to core material from Lake Tanganyika, East African Rift Valley. This lake has a well‐established palaeoenvironmental history combined with deposition of sediments in anoxic conditions allowing for ideal organic carbon preservation. Methodological refinements were made to reduce and assess any impact of species and vital effects on the resulting δ^13^C_diatom_ values.

## Study sites

### Contemporary UK river sites

**Figure 1:**
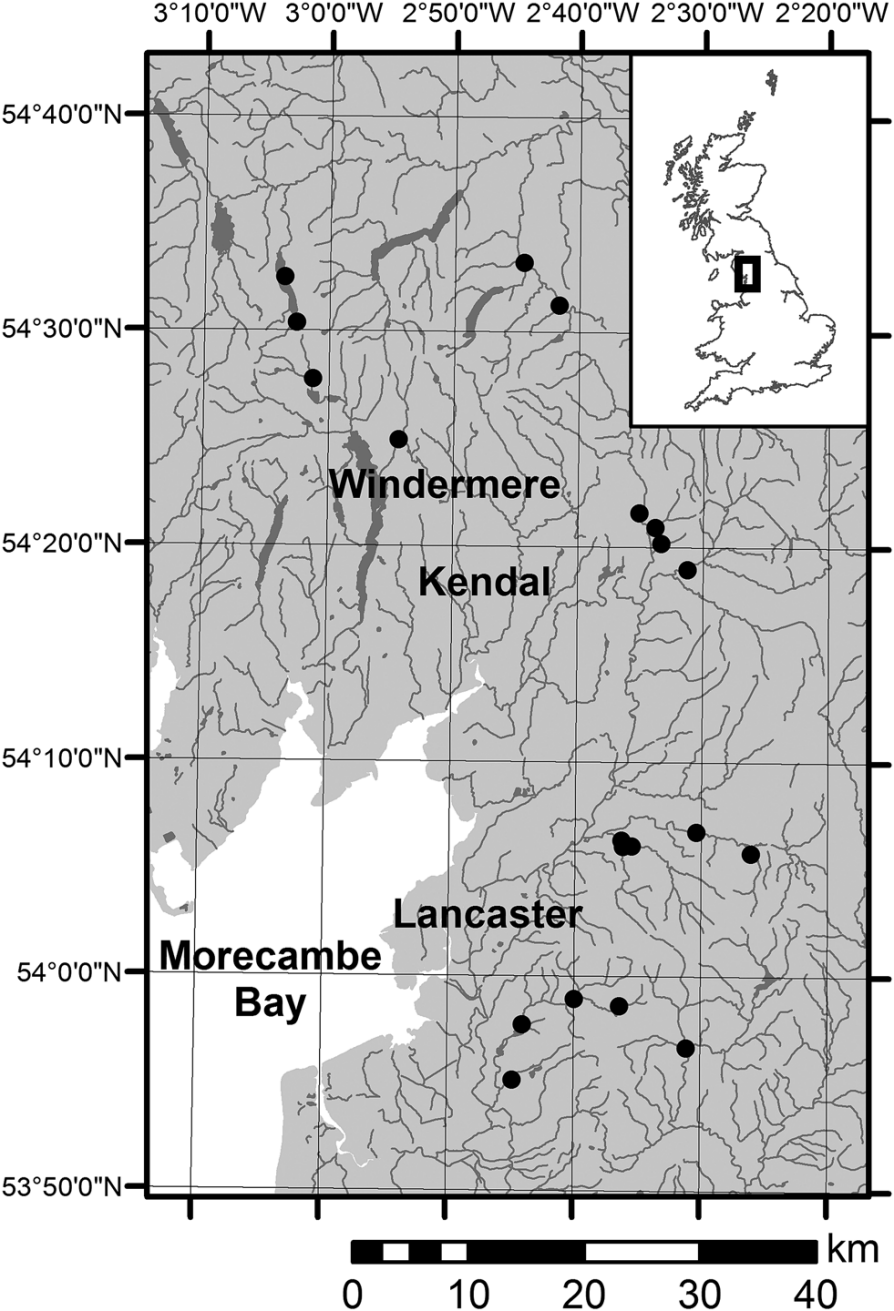
Location of the 20 UK river sites in this study. Lakes and watercourses are also shown.

Sampling of the north‐west England river catchments for epilithon and spot water chemistry samples took place in May 2012 over consecutive days to minimize hydroclimate variability. Collection from riffles in 20 river reaches captured the late spring diatom bloom. Six major river catchments were targeted: the Wyre, Ribble, Lune, Derwent, Leven and Eden, situated in North Lancashire and Cumbria (Fig. 1). Land use was largely agricultural with land proximal to the river and stream sites dominated by rough and improved pasture for grazing. The geology underlying the sampling sites consisted of combinations of sedimentary rocks (sandstones, siltstones, mudstones and gritstones) with volcanic rocks at the most easterly sites.

### Contemporary European lake sites

**Figure 2:**
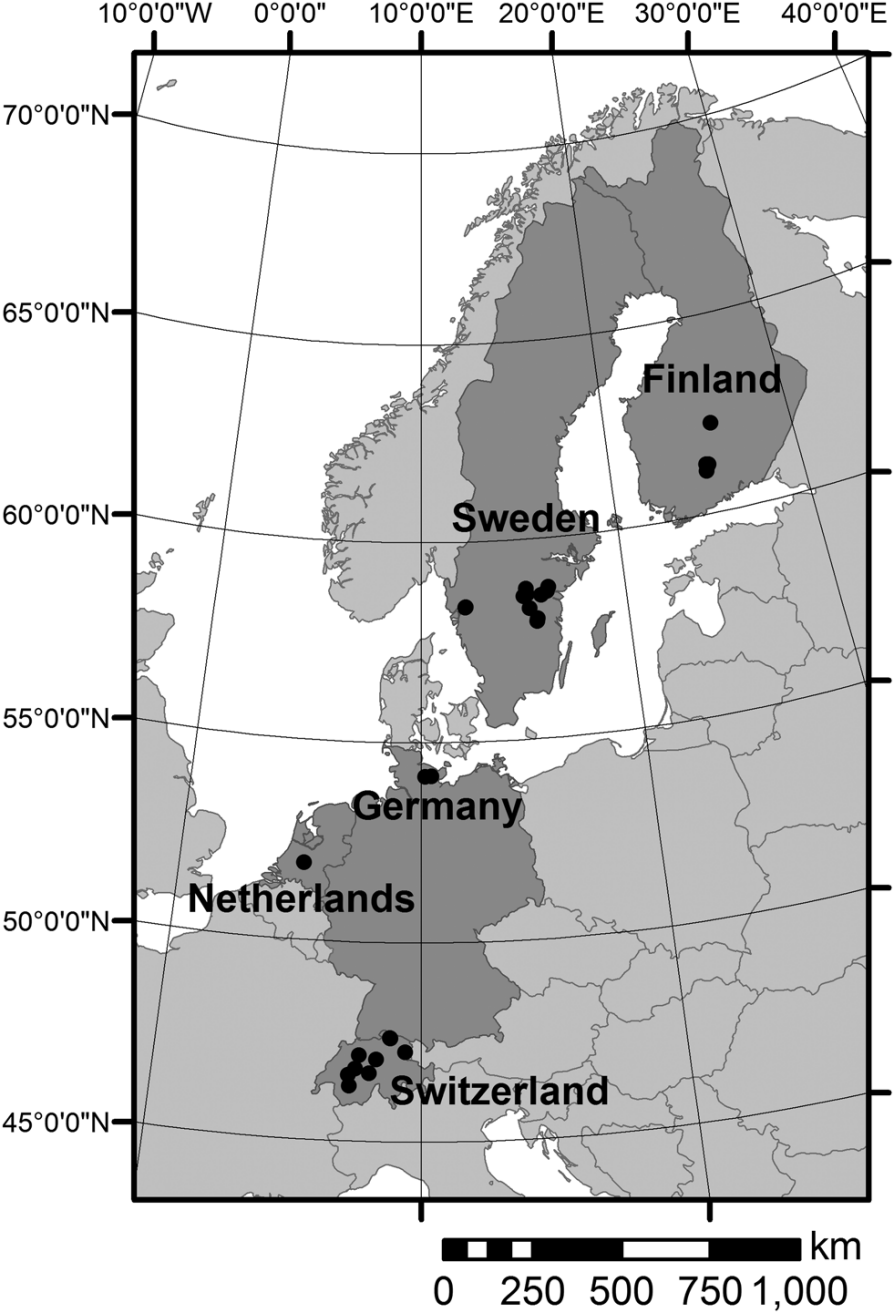
Location of the 30 European lake sites in this study.

The European lake study consisted of surface sediments analogous to core tops, and spot water chemistry samples from 30 relatively small (0.3–303 ha) lakes situated in five countries; Switzerland, the Netherlands, Germany, Sweden and Finland (Fig. 2). Water samples were collected 0.5 m below the surface in late summer before the breakdown of water stratification in the autumn (Rinta *et al*., 2015). The Swiss, Dutch and German lakes are located in the temperate zone, whereas the Finnish and Swedish lakes are in the hemiboreal to boreal zones. The underlying lithology of the Swiss, Dutch and German lakes is dominated by Quaternary sediments and older limestones. In contrast, most of the Finnish and Swedish lakes are underlain by non‐calcareous Precambrian bedrock covered by Quaternary deposits.

### Lake Tanganyika sediments

**Figure 3:**
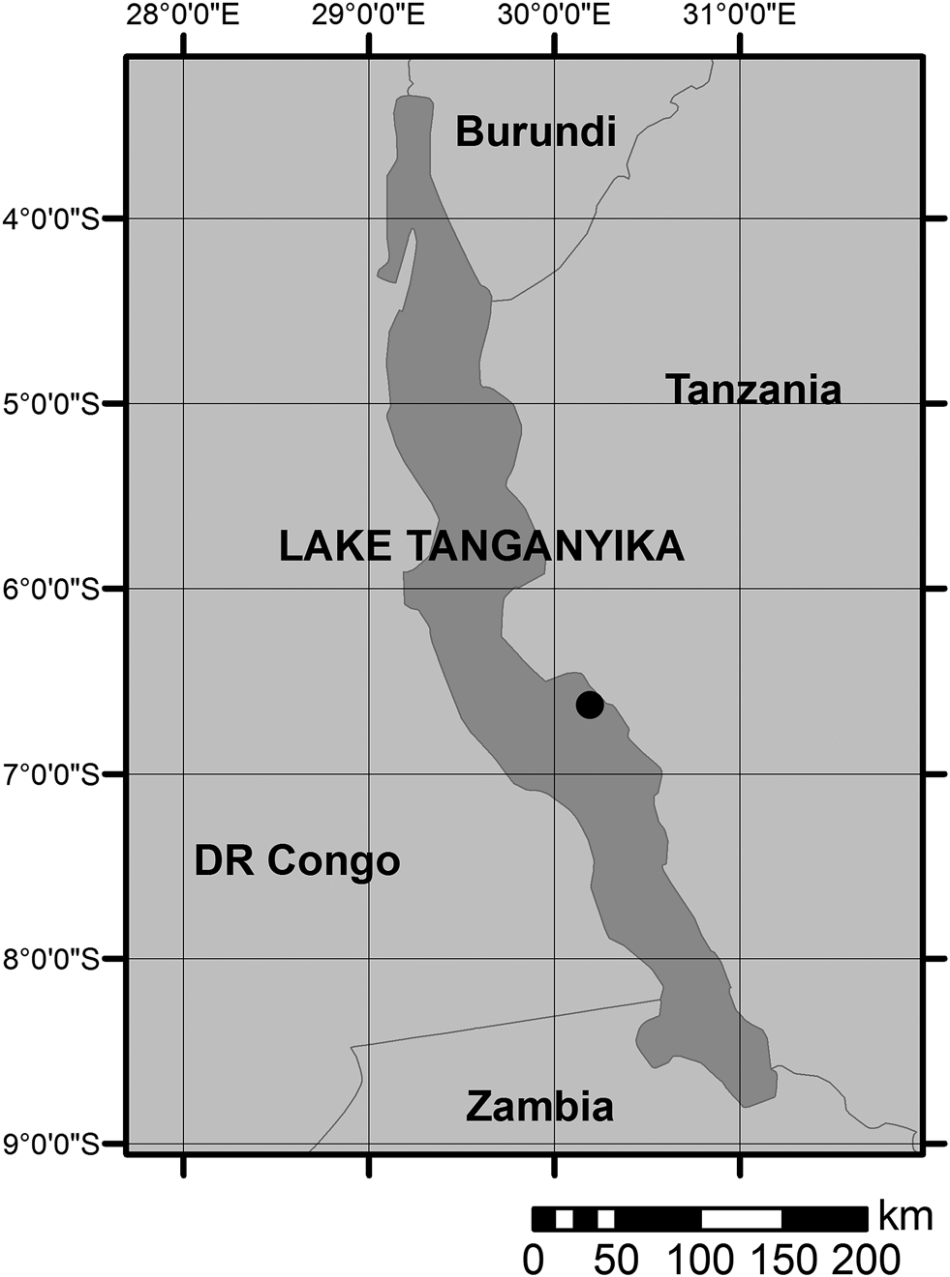
Location of the coring site in Lake Tanganyika, situated in the East African Rift Valley.

The Lake Tanganyika down‐core study comprised 14 sediment samples taken from Kullenberg piston core NP04‐KH04‐4A‐1K collected in 2004 as part of the Nyanza Project (Felton *et al*., 2007). The pelagic zone of meromictic Lake Tanganyika is highly sensitive to catchment changes that alter the carbon and nutrient concentrations. The core was taken from the Kalya Horst, which is a structural high within the southern basin of Lake Tanganyika (Fig. 3). The coring location was situated below the oxycline, the anoxic state providing ideal conditions for organic carbon preservation. Sediments were dated to the last ca. 34 000 years by correlation to a second, directly radiocarbon‐dated core (NP04‐KH04‐3A‐1K) (Tierney *et al*., 2008) using 20 age/depth control points.

## Methods

### Pretreatment and measurement of δ^13^C_diatom_

Successful determination of δ^13^C_diatom_ relies on the removal of all sources of both inorganic and organic carbon external to the frustule inclusions. To produce clean diatom material from the samples with variable organic carbon content we adapted the method described by Hurrell *et al*. (2011), which was based on that of Singer and Shemesh (1995). All samples were first passed through a 1‐mm gauge sieve then heated to 70 °C in 10% HCl for 2 h to remove inorganic carbon. For the Lake Tanganyika material a sub‐sample was removed at this stage for δ^13^C_bulk_.

For the determination of δ^13^C_diatom_, organic carbon (excluding the occluded material) was removed through oxidation by heating samples in 30% H_2_O_2_ for 15 h at 70 °C and a further 2 h at 100 °C. Persistent organic carbon was eliminated through heating samples to 70 °C for 1 h in concentrated HNO_3_. Large mineral grains were separated by differential separation and discarded. Clay and silt particles with similar densities to diatoms were reduced by sieving to 20 µm. Samples with no more than 1% carbon content were considered free from contamination following Hurrell *et al*. (2011). Sieving to 20 µm was also completed to reduce potential influence of species effects attributed to cell size and geometry (Crosta and Shemesh, 2002). Permanent slides were made of the processed samples and outline diatom counts were made based on 150 valves. For the contemporary samples, diatom genera were categorized by growth habitat as planktonic, benthic or colonial after Bellinger and Sigee (2010) as a further check for possible confounding vital effects.

^13^C/^12^C ratios for diatom material and bulk organic material from sediments were determined using an Elementar vario PYRO cube elemental analyser linked to an IsoPrime100 isotope ratio mass spectrometer at Lancaster University for contemporary UK river samples and Lake Tanganyika sediments, and at Isoprime UK in Cheadle for contemporary European lake samples. Analysis was by combustion within tin capsules at 950 °C. ^13^C/^12^C ratios were corrected against VPDB using within‐run analysis of standards IAEA‐CH‐6 (sucrose), Low Organic Content Soil Standard OAS and High Organic Content Soil Standard OAS [assuming δ^13^C values of −10.45‰ (International Atomic Energy Agency, 2011), −27.46 and −26.27‰ (Elemental Microanalysis, 2011), respectively]. Data are reported in the usual delta notation; within‐run replication of standard materials was <0.2‰ (1 SD, *n* = 10). To ensure consistency between laboratories and conditions of analysis, external precision was monitored by use of a standard material analysed between all run sequences <0.2‰ (1 SD, *n* = 164). Precision of sample analysis was <0.2‰ (1 SD, *n* = 3). Where *n* = 2, sample replicates did not vary by more than 0.5‰.

### Water analysis

In‐stream spot measurements of river site pH and electrical conductivity (EC) were taken using a WTW Multi 340i multi‐parameter water meter. Measurement accuracy was to 0.03 pH units and 1 µS cm^−1^. Analysis of river water samples was completed at Lancaster University. Total phosphorus (TP) was measured following an acid‐persulphate digest (O'Dell, 1993) using a Seal Analytical AQ2+ discrete colorimetric analyser (Seal Analytical, 2005). Total dissolved nitrogen (TDN) was measured using an Analytical Sciences Thermalox analyser (BS EN, 2003). Detection limits (standard deviation of blanks multiplied by 3) for TP and TDN analysis were 0.005 and 0.13 mg L^−1^, respectively.

For determination of δ^13^C of the DIC pool (δ^13^C_DIC_) at the river sites, 10 mL of river water was injected into 12 mL pre‐evacuated exetainers containing 150 µL of de‐gassed, concentrated phosphoric acid after Waldron *et al*. (2007). δ^13^C values of the product CO_2_ were measured at the NERC Centre for Ecology and Hydrology, Lancaster, using a GV Instruments Tracegas Pre‐concentrator coupled to an IsoPrime isotope ratio mass spectrometer. The isotope ratio of the resultant CO_2_ was compared with pulses of known reference CO_2_ and expressed relative to VPDB. Data are reported in the usual delta notation; within‐run standard replication (1 SD) was better than or equal to ±0.15‰.

Lake water spot samples and measurements were taken in the deepest part of each lake basin using a 5‐litre water sampler approximately 0.5 m below the surface as described in detail by Rinta *et al*. (2015). pH and EC were measured in the field using a pHScan 2 and WTW LF 330 device with TetraCon conductivity measuring cell, respectively. TP, total nitrogen (TN) and δ^13^C_DIC_ were determined using laboratory methods as described by Rinta *et al*. (2015).

## Results

### Contemporary UK river sites

At least 90% of each assemblage consisted of the same nine benthic diatom genera. *Achnanthidium* was present in all assemblages, generally as *A*. *minutissimum*, and was typically dominant alongside *Gomphonema*, *Cocconeis* and *Cymbella* species. No systematic correlation was found between δ^13^C_diatom_ and species composition in these data.

River water pH values ranged from 6.1 to 8.5 and EC from 16 to 331 μS cm^−1^ (Table 1). The nutrient concentrations confirmed these streams to have low‐to‐moderate trophic status (EA, 1998), with several TP measurements below detection and maximum TP and TDN values of 0.052 and 1.10 mg L^−1^, respectively. This is consistent with low‐intensity farming practices that dominated the sampled area of north‐west England.

**Table 1:** Spot sample water chemistry and δ^13^C_diatom_ values determined for each of the 20 UK river sites in this study. Median, maximum, minimum, range and the standard deviation for each parameter are included

Catchment	Stream/river	pH	EC (μS cm^−1^)	TP (mg L^−1^)	TDN (mg L^−1^)	δ^13^C_DIC_ (‰ VPDB)	δ^13^C_diatom_ (‰ VPDB)
Ribble/Wyre	Grizedale Brook	6.8	186	0.013	0.90	−11.0	−27.8
	River Dunsop	7.8	127	0.010	0.38	−7.4	−26.3
	River Wyre	7.8	174	0.052	0.76	−8.6	−28.4
	Marshaw Wyre	7.6	97	0.004	0.36	−7.5	−27.4
	Tarbrook Wyre	7.8	77	0.009	0.65	−7.0	−27.8
Lune South	Keasden Beck	7.9	197	0.013	0.39	−5.9	−27.3
	River Roeburn	7.9	118	0.007	0.39	−5.4	−26.2
	River Hindburn	8.0	161	0.011	0.59	−6.5	−28.2
	River Hindburn	8.1	148	0.012	0.48	−6.9	−26.5
	River Wenning	8.5	331	0.026	1.10	−8.5	−28.6
Lune North	River Rawthey	7.5	177	<0.005	0.51	−8.0	−27.3
	Crossdale beck	6.5	79	0.010	0.33	−6.9	−27.8
	Chapel beck	7.0	91	<0.005	0.39	−6.2	−25.3
	River Lune	7.8	227	0.006	0.57	−7.9	−27.4
Derwent/Levern	Trout Beck	6.6	103	<0.005	0.67	−11.5	−28.7
	River Rothay	6.1	16	0.005	0.63	−9.3	−28.3
	Unnamed	6.4	47	<0.005	0.44	−6.1	−27.8
	Unnamed	6.7	58	<0.005	0.30	−2.9	−26.0
Eden	River Lowther	7.7	200	0.006	0.61	−8.5	−28.4
	River Lowther	8.2	192	0.007	0.59	−8.0	−28.9
Median		7.7	138	n/a	0.54	−7.5	−27.8
Max.		8.5	331	0.052	1.10	−2.9	−25.3
Min.		6.1	16	<0.005	0.30	−11.5	−28.9
Range		2.3	315	n/a	0.80	8.5	3.6
SD		0.7	74	n/a	0.20	1.9	1.0

**Figure 4:**
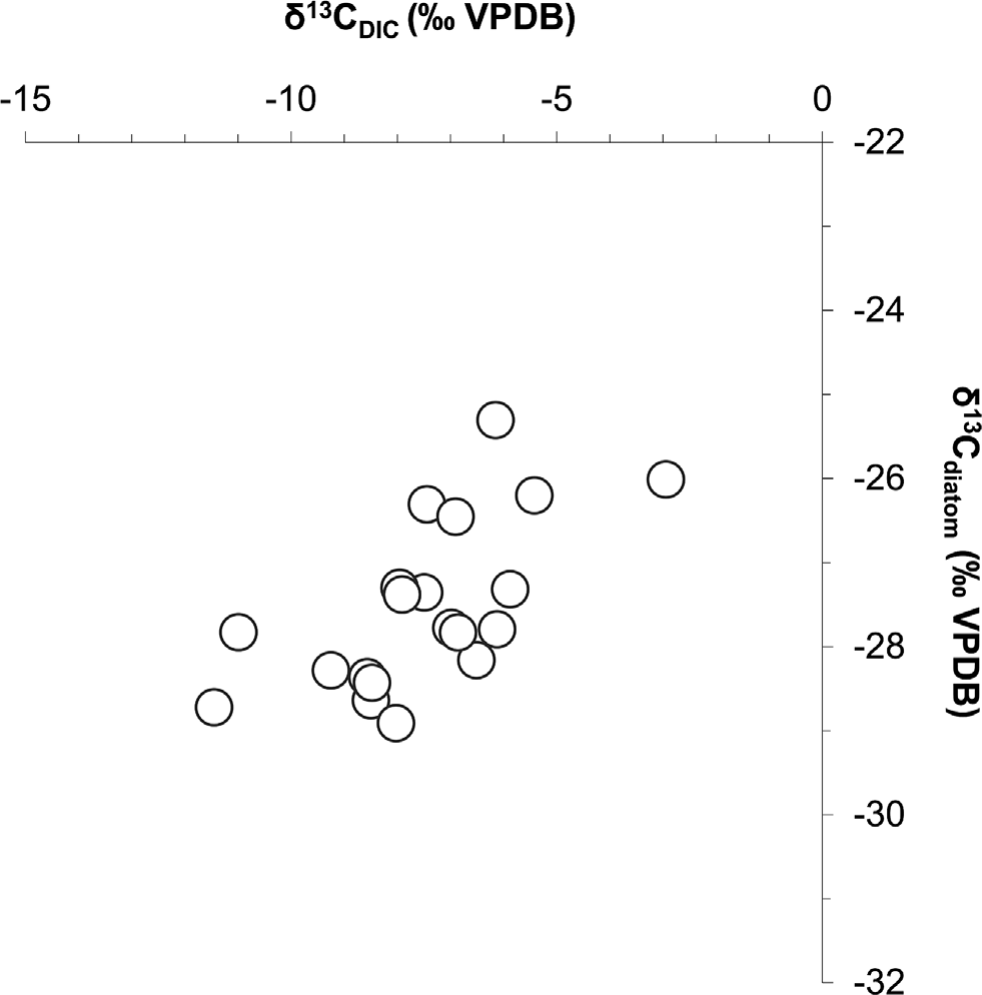
Significant positive relationship between δ^13^C_DIC_ and δ^13^C_diatom_ values determined for the contemporary UK river data set (*r_s_* = 0.70, *P* < 0.01). Relationship identified using Spearman's rank correlation coefficient.

**Figure 5:**
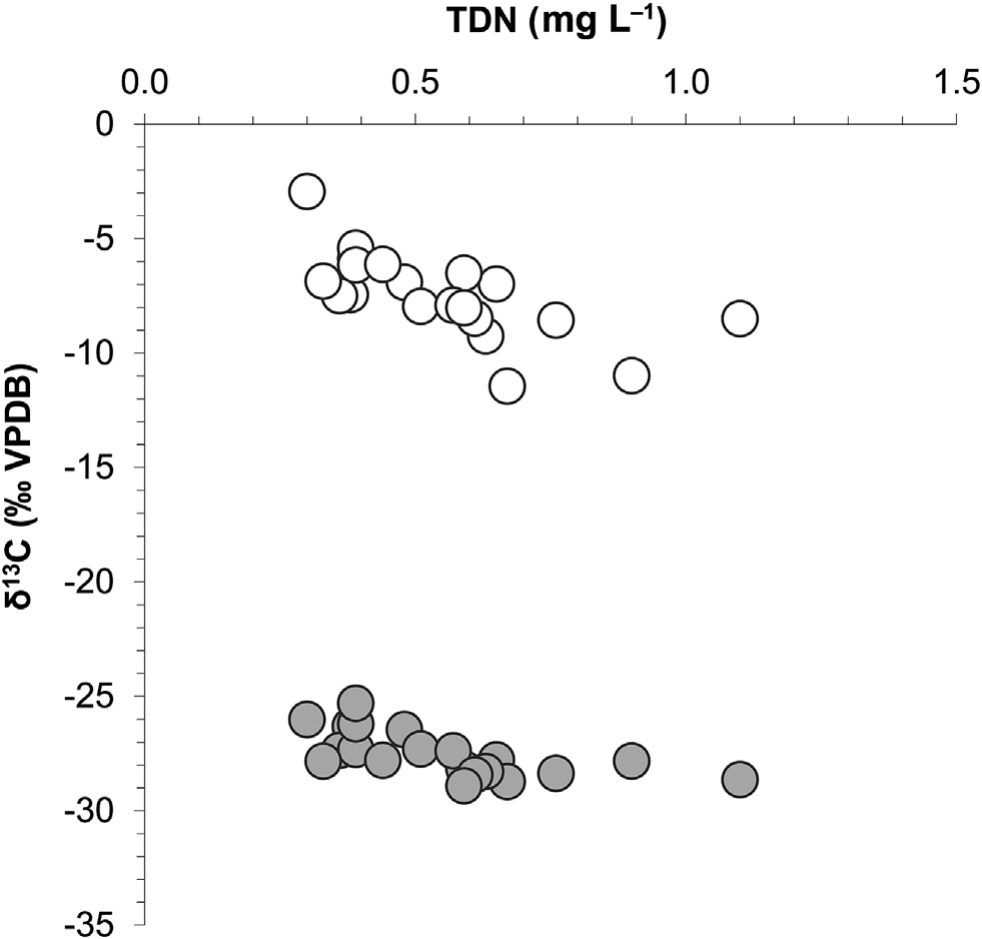
Significant negative relationships between TDN concentrations and δ^13^C_DIC_ (open circles) (*r_s_* = −0.77, *P* < 0.01) and δ^13^C_diatom_ (shaded circles) (*r_s_* = −0.73, *P* < 0.01) values determined for the contemporary UK river data set. Relationships identified using Spearman's rank correlation coefficient.

δ^13^C_DIC_ of the UK river waters ranged between −11.5 and −2.9‰. In contrast, δ^13^C_diatom_ had a smaller range, lying between −28.9 and −25.3‰. Correlation analysis using Spearman's rank correlation coefficient (IBM SPSS) identified a significant positive relationship between δ^13^C_DIC_ and δ^13^C_diatom_ (*r_s_* = 0.70, *P* < 0.01) (Fig. 4). Significant negative relationships were also identified between TDN concentrations and δ^13^C_DIC_ (*r_s_* = −0.77, *P* < 0.01) in addition to TDN concentrations and δ^13^C_diatom_ (*r_s_* = −0.73, *P* < 0.01) (Fig. 5).

### Contemporary European lake sites

The lake sediment diatom assemblages comprise planktonic life forms alongside benthic and colonial examples making them more diverse than the river assemblages. Dominant genera included planktonic *Cyclotella*, *Aulacoseira* and *Stephanodiscus* species. Despite this diversity, no systematic correlation was found between δ^13^C_diatom_, the species data or the proportions of different life forms.

In comparison with the UK river study set, larger ranges in water chemistry values were measured in the lake waters. The pH values ranged between 5.4 and 8.9 and EC values from 24 to 462 μS cm^−1^ (Table 2). Trophic conditions varied from ultra‐oligotrophic to hypertrophic (OECD, 1982), reflecting a wide range of nutrients with maximum TP and TN values of 0.12 and 2.30 mg L^−1^, respectively.

**Table 2:** Spot sample water chemistry and δ^13^C_diatom_ values determined for each of the 30 European lake sites in this study. Median, maximum, minimum, range and the standard deviation for each parameter are included. Data from Rinta *et al*. (2015)

Country	Lake	pH	EC (μS cm^−1^)	TP (mg L^−1^)	TN (mg L^−1^)	δ^13^C_DIC_ (‰ VPDB)	δ^13^C_diatom_ (‰ VPDB)
Switzerland	Lauenensee	6.6	462	0.007	0.53	−7.9	−26.1
	Schwarzsee	6.9	386	0.015	0.42	−9.0	−25.8
	Hinterburgsee	8.9	137	0.011	0.80	−6.0	−27.5
	Gerzensee	7.0	289	0.016	0.68	−4.4	−27.7
	Rotsee	8.8	183	0.034	0.81	−5.4	−27.1
	Burgäschisee	8.6	275	0.014	1.20	−5.6	−27.3
	Seealpsee	8.7	150	0.010	0.55	−6.6	−25.8
	Hasensee	7.7	329	0.036	1.20	−5.1	−27.5
	Hüttwilersee	8.5	316	0.015	1.10	−4.3	−26.7
	Nussbaumersee	8.1	346	0.024	1.20	−6.6	−26.7
Netherlands	De Waay	8.0	368	0.120	2.30	−9.1	−26.7
Germany	Holzsee	8.2	328	0.036	0.86	−5.5	−26.5
	Schöhsee	8.2	247	0.017	0.57	−2.7	−25.4
Sweden	Glimmingen	6.9	57	0.008	0.33	−19.0	−26.6
	Kisasjön	7.7	127	0.018	0.49	−18.9	−27.7
	Hargsjön	6.7	214	0.045	1.17	−19.3	−28.6
	Skottenesjön	6.9	139	0.038	0.65	−20.3	−27.5
	Erssjön	6.3	55	0.018	0.66	−19.7	−29.3
	Illersjön	7.6	311	0.025	0.48	−19.6	−27.8
	Mårn	7.7	132	0.027	1.05	−19.7	−26.5
	Storå vänstern	5.7	78	0.009	0.46	−19.3	−27.7
	Lillsjön	7.0	41	0.018	0.61	−20.1	−31.1
	Skärgölen	7.8	48	0.012	0.35	−18.4	−27.9
	Grissjön	6.5	27	0.011	0.39	−18.9	−29.9
Finland	Lovojärvi	7.1	123	0.027	0.84	−13.4	−26.5
	Syrjänalunen	6.1	59	0.003	0.20	−21.6	−29.4
	Nimetön	5.6	68	0.010	0.48	−23.6	−33.4
	Mekkojärvi	5.4	44	0.011	0.61	−14.0	−26.9
	Valkea‐Kotinen	5.9	24	0.011	0.57	−22.8	−30.4
	Jyväsjärvi	6.1	75	0.025	0.62	−17.6	−26.1
Median		7.1	138	0.017	0.62	−15.8	−27.4
Max.		8.9	462	0.120	2.30	−2.7	−25.4
Min.		5.4	24	0.003	0.20	−23.6	−33.4
Range		3.5	439	0.117	2.10	20.9	8.0
SD		1.0	130	0.021	0.41	7.0	1.8

δ^13^C_DIC_ values varied from −23.6 to −2.7‰, and two distinct clusters were observed: the Swiss, Dutch and German lakes had δ^13^C_DIC_ > −10‰ (group 1), and the Swedish and Finnish lakes δ^13^C_DIC_ < −10‰ (group 2). These groupings corresponded to differences in catchment lithology, with lakes situated in hard water catchments containing calcareous bedrock (group 1) associated with δ^13^C_DIC_ > −10‰.

The range in δ^13^C_diatom_ values from −33.4 to −25.4‰ is lower than the range in δ^13^C_DIC_. Statistical comparison using a Mann–Whitney test (IBM SPSS) found the δ^13^C_diatom_ values of each group of lakes differed significantly (*U* = 42.5, *z* = −2.85, *P* < 0.01, *r* = −0.52). Generally, lakes with calcareous catchments (group 1) had more positive δ^13^C_diatom_ (median: −26.7‰) compared with lakes in group 2 situated in non‐calcareous catchments (median: −27.8‰). In addition a smaller range in values of 2.3‰ was present in group 1 compared with 7.3‰ in group 2.

**Figure 6:**
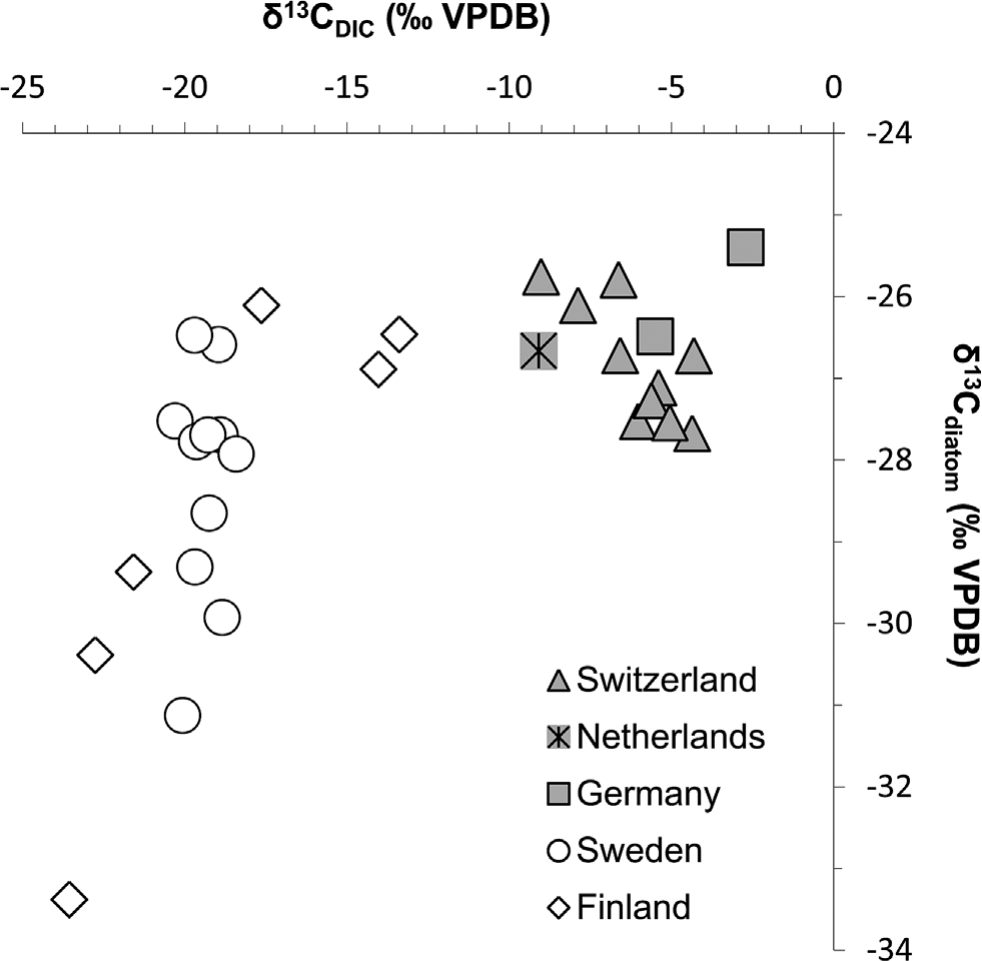
Plot of contemporary European lake data set δ^13^C_DIC_ and δ^13^C_diatom_ values. A significant positive relationship was present across the whole data set (*r_s_* = 0.59, *P* < 0.01). When split and re‐analysed no significant relationship was present between group 1 data (shaded symbols) (*r_s_* = −0.25, *P* = 0.41), but a stronger relationship was seen within group 2 (open symbols) (*r_s_* = 0.63, *P* < 0.01). Relationships identified using Spearman's rank correlation coefficient.

As seen within the UK river data, a significant positive relationship was present between European lake δ^13^C_DIC_ and δ^13^C_diatom_ (*r_s_* = 0.59, *P* < 0.01) (Fig. 6). As was found for δ^13^C_DIC_, two groupings of δ^13^C_diatom_ values emerged, with the Swiss, Dutch and German data (group 1) forming a cluster of higher isotope values, and the Swedish and Finnish data points (group 2) spread along a linear gradient of lower isotope values. There was a strong relationship between δ^13^C_DIC_ and δ^13^C_diatom_ in group 2 (*r_s_* = 0.63, *P* < 0.01) but not between the equivalent values for lakes in group 1 (*r_s_* = −0.25, *P* = 0.41). No significant relationships were identified between δ^13^C_diatom_ and either TP (*r_s_* = 0.13, *P* = 0.49) or TN (*r_s_* = 0.23, *P* = 0.22) concentrations.

### Lake Tanganyika sediments

**Figure 7:**
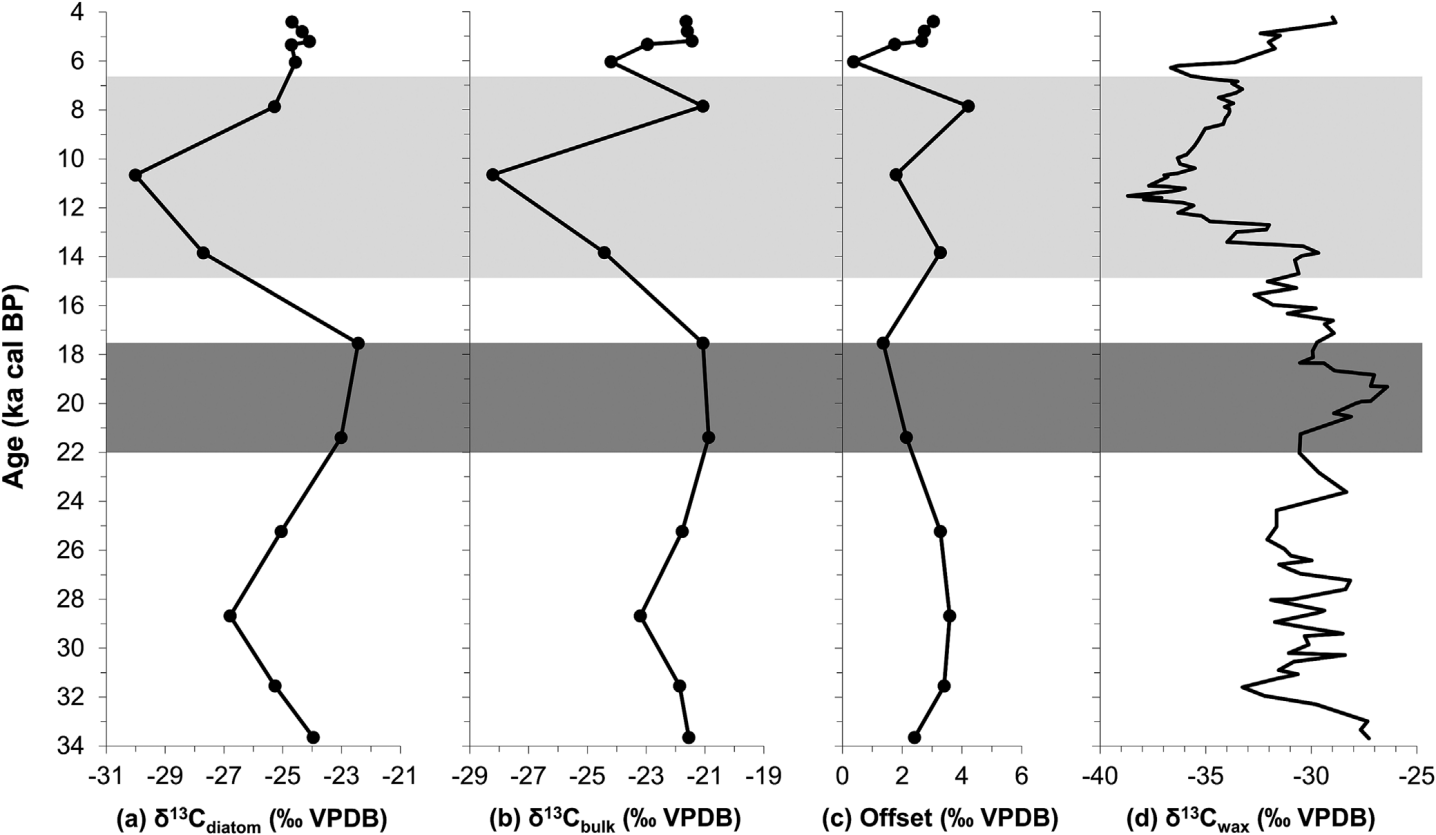
δ^13^C values from Lake Tanganyika sediments plotted against sample age. δ^13^C_diatom_ (a), δ^13^C_bulk_ values (b) and the offset between the two records (c) were determined for Lake Tanganyika sediment core NP04‐KH04‐4A‐1K. δ^13^C_wax_ data are re‐plotted from Tierney *et al*. (2010). Pale grey shading represents the African Humid Period as defined by deMenocal *et al*., (2000). Dark grey shading represents the likely peak in arid conditions experienced in East Africa during the last glacial (Barker and Gasse, 2003).

Diatom communities reconstructed from the sediments were dominated by planktonic taxa including *Cyclotella*, *Aulacoseira* and *Stephanodiscus* species. δ^13^C_diatom_ varied from −30.0 to −22.4‰, equating to a range of 7.6‰ (Fig. 7a). In comparison, δ^13^C_bulk_ values were higher with a range of 7.3‰ from −28.2 to −20.9‰ (Fig. 7b). The offset between the two data sets varied from 0.4 to 4.2‰ with a median value of 2.7‰ (Fig. 7c). A significant positive relationship was present between δ^13^C_diatom_ and δ^13^C_bulk_ (*r_s_* = 0.73, *P* < 0.01). At this coarse millennial scale, lowest values for both δ^13^C_diatom_ and δ^13^C_bulk_ occurred between 14.8 and 5.5 ka, the period broadly recognized as the African Humid Period (deMenocal *et al*., 2000) (light shading in Fig. 7). Conversely, both isotope proxies had their maximum values in sediments dating to the end of the last glacial period and again in the late Holocene. Here also the smallest offset between the two records was measured. The corresponding trends in δ^13^C_diatom_ and δ^13^C_bulk_ closely track that of higher plant leaf waxes (δ^13^C_wax_) (Fig. 7d), a terrestrial vegetation change proxy extracted from Lake Tanganyika sediments by Tierney *et al*. (2010).

## Discussion

Previous studies of freshwater environments have identified (i) δ^13^C of carbon sources, (ii) the relative contributions of these sources and (iii) ^13^C enrichment by preferential ^12^C uptake by lake primary producers (including diatoms) as key variables determining the δ^13^C_DIC_ available to diatoms for assimilation as δ^13^C_diatom_. Investigation into environmental controls of contemporary δ^13^C_diatom_ over the different spatial and temporal scales reported here pinpoints the relative influence of carbon supply and demand factors, aiding the interpretation of palaeolimnological records as well as contemporary carbon cycling.

### Translation of the carbon cycling history

Positive relationships identified between δ^13^C_DIC_ from waters and contemporary δ^13^C_diatom_ represent the primary control of catchment carbon source on the carbon isotopes in the diatom frustules. Principal carbon sources within a freshwater catchment have both geological and biotic origins and are associated with differing carbon isotopic ranges. Weathering of calcareous rock releases bicarbonate, which has a relatively high δ^13^C value of 0 to +1‰ (Clark and Fritz, 1997). In contrast, CO_2_ released into soils from plant root respiration and vegetation decay has a lower δ^13^C value, ranging from −26 to −20‰ in C_3_ and −12 to −6‰ in C_4_ landscapes, respectively (Mook *et al*., 1974; O'Leary, 1988). Oxidation of methane, associated with δ^13^C values between –80‰ and –50‰, provides a carbon source that is even further ^13^C depleted (Whiticar, 1999).

These catchment carbon signatures are transported via infiltrating flows to freshwater bodies where they mix with dissolved carbon from autochthonous sources, including macrophytes with recorded δ^13^C values of −50 to −11‰ (Keeley and Sandquist, 1992) and phytoplankton with bulk values ranging between −42 and −26‰ (Leng and Marshall, 2004). Oxidation of such materials during decomposition enables further release of ^13^C depleted CO_2_, but if waters are stratified this process is slowed and the potential biotic carbon source is stored within anoxic sediments (Leng and Marshall, 2004).

The resulting δ^13^C_DIC_ signature of a water body is further impacted by atmospheric exchange leading to the preferential loss of ^12^C. In addition, primary productivity (including diatoms) results in discriminatory uptake of ^12^C. The pH of a water body is also significant as it determines the proportioning of different DIC species; each of which has a contrasting carbon isotope signature. At pH 8 the percentage of DIC present as dissolved CO_2_ is close to 0, and the hydration and disassociation of dissolved CO_2_ into bicarbonate causes an increase in δ^13^C of approximately 9‰ (Clark and Fritz, 1997).

δ^13^C_DIC_ is therefore a record of catchment carbon cycling history reflecting sourcing and further changes to the isotope value related to fractionation during carbon phase and species changes. Final translation of the δ^13^C_DIC_ signature to δ^13^C_diatom_ is dependent on the photosynthetic pathway used, and any species‐specific or vital effects that determine the degree of fractionation on uptake. The positive correlation identified between δ^13^C_DIC_ and δ^13^C_diatom_ in the UK rivers (Fig. 4) and European lakes (Fig. 6) suggests that even in sites of highly varying environmental characteristics, δ^13^C_DIC_ is a significant control in the determination of δ^13^C_diatom_.

### The role of catchment productivity

Within the contemporary UK rivers sampled, DIC probably had a biotic origin as no significant areas of calcareous geology were present in any of the catchments. The negative relationship identified between δ^13^C_DIC_ and TDN concentrations (Fig. 5) supports this conclusion and indicated that river DIC pool characteristics were probably controlled by catchment productivity at this scale. This relationship showed that dissolved biotic carbon supply was enhanced within more productive catchments and aquatic primary productivity did not lead to relative enrichment in ^13^C. This agrees with the findings of Maberly *et al*. (2013) who found a greater availability of DIC associated with more productive catchments in the English Lake District. Increased loadings of dissolved biogenic carbon with a relatively low δ^13^C signature were attributed to catchment land use, with greater availability of nutrients resulting in enhanced dissolved carbon release within the catchment.

The absence of a correlation between δ^13^C_diatom_ and species composition suggests sieving to control for species‐specific and vital effects was successful, or at least limited any significant impact on diatom isotopic value. In addition δ^13^C_diatom_ values were consistently lower than δ^13^C_DIC_ and fell within a small range of 3.6‰. This suggests possible uptake of bicarbonate via CCMs has not had a pronounced effect on the resulting diatom carbon values. These findings show that δ^13^C_diatom_ can be used to investigate catchment productivity, highlighting the close coupling between a water body and its catchment conceptualized as the balance between carbon supply and demand. For specific catchments the strength of this relationship is dependent on the multiple and interacting controls on δ^13^C_DIC_ in lake waters and its translation to the diatoms.

### The role of catchment geology

In comparison with the UK river epilithon, the European lake sediments represented a potentially more integrated temporal record of carbon cycling, with seasonal variability masked by sediment accumulation during several annual cycles. In addition, the much broader geographical range was clearly manifest in the heterogeneity of water chemistry variables and diatom assemblage compared with that of the rivers. Most striking was the identification of two groupings in δ^13^C_diatom_ values, which coincided with both δ^13^C_DIC_ and major geological differences in catchment carbon source characteristics.

At the continental scale of analysis, no relationships were identified between δ^13^C_diatom_ and productivity indicators TP or TN, either in the complete data set or within sub‐groups. It is likely that highly varied catchment carbon processing attributed to the diverse climate, land use and geology represented obscured any record of pelagic carbon demand differences between the lakes. As seen with the UK rivers, the impact of species effects on δ^13^C_diatom_ appears to have been limited successfully through sieving to <20 µm. Instead, DIC characteristics appear to be the principal environmental controls of δ^13^C_diatom_ at this scale. Higher δ^13^C_diatom_ signatures (−27.7 to −25.4‰) in lakes with calcareous catchments (group 1) compared with those without (−33.4 to −26.1‰) (group 2) reflected the contribution of carbonate geology (associated with δ^13^C between 0 and +1‰) to respective lake carbon pools. This demonstrated the significance of DIC sourcing to the production of an initial carbon signature, which is transferred to δ^13^C_diatom_. Also influential was the relative availability of DIC for uptake by diatoms. The absence of a relationship between δ^13^C_DIC_ and δ^13^C_diatom_ in group 1 is probably a result of high background levels of geologically sourced dissolved carbon. In addition, the influence of geological sourcing on δ^13^C_diatom_ in group 1 may have been compounded by likely enhanced uptake of bicarbonate via CCMs due to the near 0% contribution of CO_2_ to DIC at pH values over 8. It is only in the absence of significant geological carbon sources (group 2) where the transfer of a dissolved biogenic carbon signature reflecting catchment productivity can be determined in δ^13^C_diatom_.

### Interpretation of palaeoenvironmental records

Advancement of the findings by Maberly *et al*. (2013) concerning lake catchment productivity to include rivers has important implications for interpretation of palaeoenvironmental records. The close coupling between freshwater networks and catchment carbon cycling, and in particular the relative availability of dissolved biogenic carbon in response to land use, has been clearly demonstrated. Analysis of δ^13^C_diatom_ from highly varied lake sites demonstrated the difficulties associated with developing a universal model of catchment and water productivity relationships. Nevertheless, successful extraction of δ^13^C_diatom_ from lake sediments highlights the potential for obtaining palaeoenvironmental archives of changes in catchment carbon cycles from lake sediment cores. In particular, the fundamental principal of a carbon supply and demand balance can be applied to the interpretation of freshwater δ^13^C_diatom_ extracted from lakes situated in contrasting environmental and climatic settings.

The sediments from Lake Tanganyika provided an opportunity to test these conclusions on a lake with a well‐established palaeoenvironmental history (e.g. Gasse *et al*., 1989; Scholz *et al*., 2003; Talbot *et al*., 2006) that would be expected to respond to changes in carbon cycling at the landscape scale. Within the Lake Tanganyika sediments there is a close coupling between δ^13^C_diatom_ and δ^13^C_bulk_ throughout the 34 000‐year record with an offset no greater than 4.2‰ (Fig. 7). Diatom values were consistently lower than bulk carbon, suggesting diatoms were using the lighter isotope from dissolved carbon inputs. Using the conceptual relationships developed above, these isotope changes are thought to indicate that the lake carbon pool principally reflected changes in the quantity and nature of carbon supplied from the catchment with modifications by lake primary productivity as a secondary factor. This finding is attributed to the great size of Lake Tanganyika and its catchment where, particularly during wet periods, dissolved and particulate biogenic carbon produced in the catchment would have significantly contributed to the lake carbon pool.

Even during dry intervals of the last glacial period (Barker and Gasse, 2003) and the late Holocene (Haberyan and Hecky, 1987), signified by high δ^13^C signatures in both δ^13^C_diatom_ (−24.7 to −22.4‰) and δ^13^C_bulk_ (−21.6 to −20.9‰), maintenance of a correlation and a constant offset indicates primary productivity did not significantly deplete the carbon pool. This is despite a probable decrease in carbon delivery from the catchment and potential enhancement of lake mixing processes leading to nutrient recycling (Scholz *et al*., 2003). In addition to variability in carbon loading, the coinciding measurement of high δ^13^C signatures for diatoms and bulk sediments suggests a change in carbon source. Corresponding high δ^13^C_wax_ values (−29.0 to −26.4‰) at this time (Fig. 7d) indicate increased prevalence of C_4_‐dominated savanna grassland within the Lake Tanganyika catchment (Tierney *et al*., 2010). Because of these ecosystem changes, greater contributions of dissolved carbon with higher δ^13^C entered the lake during dry periods, and were thus translated into higher δ^13^C_diatom_ and δ^13^C_bulk_ values. The discovery of pervasive terrestrial supply domination over aquatic demand suggests large lakes are likely to have been substantial carbon sources to the atmosphere over centennial to millennial timescales. This contrasts with smaller lakes such as Lake Challa on the flank of Kilimanjaro where diatom and bulk carbon isotope records became periodically decoupled by enhanced in‐lake productivity (Barker *et al*., 2013).

The Lake Tanganyika study demonstrates that comparison of δ^13^C_diatom_ with δ^13^C_bulk_ extracted from sediment cores enables catchment and lake carbon cycles to be disentangled, overcoming inherent ambiguities in the interpretation of bulk δ^13^C. If geological carbon sources of lakes can be assumed to be constant and modifications to the soil carbon pool from vegetation changes can be understood, individual site histories can be reconstructed. Lake sediment δ^13^C_diatom_ therefore represents a largely under‐exploited resource with the potential to provide highly insightful carbon cycling chronologies over millennial timescales.

## Conclusions

Stable isotope analysis of diatom organic molecules, occluded in silica, constrains uncertainties associated with measurements of undifferentiated sedimentary carbon. The occluded organic matter also provides a carbon archive largely protected from degradation, oxidation and diagenesis. The application of the δ^13^C_diatom_ method in freshwaters requires adjustments to the standard inferences concerning pelagic productivity established by early marine studies. In freshwaters the controls of δ^13^C_diatom_ are more complex due to the high degree of connectivity between terrestrial vegetation, soils, bedrock and aquatic ecosystems. Within these environments carbon supply‐side characteristics, including relative abundance of DIC from differing sources and associated δ^13^C_DIC_, are important controls that change as a function of catchment characteristics at various spatial scales.

The concept of inorganic carbon supply and demand offers a useful framework through which to develop the interpretation of δ^13^C_diatom_. Analysis of contemporary δ^13^C_diatom_ from UK river epilithon demonstrates the close linkages between carbon cycling in freshwater networks and their catchments. In support of findings by Maberly *et al*. (2013), more productive catchments are associated with greater availability of dissolved biogenic carbon. Investigation of contemporary European lake surface sediments confirms palaeolimnological inferences concerning catchment control of lake carbon supply made by Barker *et al*. (2013) and Hernández *et al*. (2013, 2011). Consequently variations within a single site may be readily interpreted in terms of land use as the lithological template is held constant. It is presently not possible to produce a globally relevant quantitative relationship between δ^13^C_diatom_ and specific environmental variables. However, as demonstrated by the Lake Tanganyika study, great potential lies in the use of δ^13^C_diatom_ to inform interpretation of lake sediment records. Palaeoenvironmental interpretation could be further enhanced by modelling the transfer of carbon through specific catchments. Of particular significance is improved understanding of dissolved carbon cycling from diatom frustules, independent of particulate carbon compositional changes normally associated with lake sediment δ^13^C_bulk_ analysis, to evaluate changes in freshwater ecosystems and palaeoenvironments.
